# Solving Riddles Through Sequencing (SIRIUS): unlocking hematologic diagnoses by whole genome and transcriptome sequencing

**DOI:** 10.1038/s41375-025-02820-2

**Published:** 2025-12-19

**Authors:** Marietta Truger, Manja Meggendorfer, Wencke Walter, Stephan Hutter, Winfried Alsdorf, Gero Massenkeil, Uwe M. Martens, Wolfgang Kern, Katharina Hörst, Constanze Kühn, Andreas Reiter, Andreas Hochhaus, Torsten Haferlach

**Affiliations:** 1 MLL Munich Leukemia Laboratory, Munich, Germany; 2 University Medical Center Hamburg-Eppendorf, Hamburg, Germany; 3 Department of Internal Medicine II, Klinikum Gütersloh, Gütersloh, Germany; 4 Department for Hematology and Oncology, SLK-Clinics Heilbronn GmbH, MOLIT Institute for Personalized Medicine, Heilbronn, Germany; 5 Department of Hematology and Oncology, University Hospital Mannheim, Heidelberg University, Mannheim, Germany; 6 Department of Hematology and Internal Oncology, Universitätsklinikum Jena, Jena, Germany

**Keywords:** Haematological cancer, Genomics, Genetic testing, Haematological cancer

## Abstract

In the rapidly evolving field of hematology, the diagnosis of leukemias and lymphomas poses major challenges, despite significant genetic advancements. Although established diagnostic methods comprise a multidisciplinary approach and are considered gold standard, in some cases they fall short in conclusively identifying hematological neoplasms. In this context, the current SIRIUS study (NCT05046444) delves into the potential of whole genome sequencing (WGS) and whole transcriptome sequencing (WTS) to bridge diagnostic gaps. By analyzing 106 patients with an unclear diagnosis or clinical condition following gold standard diagnostics, our study demonstrates that WGS and WTS can uncover a broader range of somatic alterations, including rare single-nucleotide variants (SNVs), small copy number variations (CNVs), and aberrant gene expression patterns not detected by conventional diagnostics. WGS and WTS provided additional diagnostic insights in 25% of cases, suggesting their value not only in enhancing diagnostic accuracy but also in contributing to more informed prognostic assessments and personalized treatment strategies. Therefore, our study underscores the importance of integrating WGS and WTS into the diagnostic toolbox for hematological neoplasms. This approach promises not only to improve patient outcomes but also to do so in a manner that is both financially sustainable and ethically sound.

## Introduction

Diagnosing and classifying leukemias and lymphomas remains a major challenge, even after the latest updates from the World Health Organization (WHO) and the International Consensus Classification (ICC) in 2022 [1–3]. Diagnostic decision-making primarily relies on five key pillars: cytomorphology of peripheral blood and/or bone marrow smears, histology and immunohistochemistry of bone marrow trephine and lymph node biopsies, immunophenotyping, chromosome banding analysis (CBA) complemented by fluorescence in situ hybridization (FISH) analysis, and molecular genetics, including polymerase chain reaction (PCR) based investigations and targeted panel sequencing via next-generation sequencing (NGS) with DNA or RNA/cDNA derived from peripheral blood and/or bone marrow cells. These established gold standard diagnostics necessitate interdisciplinary collaboration of specialists, including hematologists, hematopathologists, cytogeneticists, and molecular biologists. Ideally, this interdisciplinary approach leads to diagnosis, classification, including markers for measurable residual disease (MRD), prognostic assessment and the implementation of precision medicine for the benefit of the patient. Despite optimal conditions and comprehensive diagnostics, some cases with suspected hematological neoplasm or unclear clinical findings remain unsolved, as even the broad use of established methods does not always yield conclusive results. If the patient’s clinical condition does not self-resolve (e.g., following toxic exposure), significant clinical uncertainty persists.

Our study SIRIUS (Solving Riddles Through Sequencing, NCT05046444) specifically addresses such challenging cases. Massive parallel sequencing, particularly for whole genome sequencing (WGS) and whole transcriptome sequencing (WTS), enables comprehensive and simultaneous analysis of somatic alterations, including single nucleotide variants (SNVs), small insertions and deletions (indels), structural variants (SVs), and copy number variations (CNVs) and may supplement or replace classical diagnostics. However, factors such as limited sample material and the extended turnaround time for subsequent diagnostic analyses must be considered. Therefore, this study aims to analyze how often WGS and WTS provide a definitive or improved diagnosis in challenging cases unsolved by established diagnostics. Furthermore, the gain of information of prognostic or therapeutic relevance and also the turnaround times associated with conventional diagnostics versus/plus combined WGS and WTS analysis (WGTS) will be evaluated.

## Materials and methods

### Study design and patients

Our study SIRIUS was conducted as a single laboratory prospective case-control study (clinicaltrials.gov: NCT05046444) at MLL Munich Leukemia Laboratory. Between 07/2021 and 11/2024, 106 patients were recruited from 56 different sites in Germany and one from Sweden with a suspected hematological neoplasm and an unclear diagnosis or clinical condition after a broad spectrum of standard routine diagnostics. All patients or their respective legal guardians gave specific and written informed consent for the use of material and data for this study. The study adhered to the tenets of the Declaration of Helsinki and the MLL internal study board.

### Standard diagnostic procedures

Gold standard diagnostic procedures including cytomorphology, immunophenotyping, cytogenetics and molecular genetics were performed as previously described [4–6]. Only if no definitive diagnosis could be made and the results of established methods failed to explain hematological abnormalities, participation in the SIRIUS study was offered. For some cases, additional material was provided, but in most cases, left-over material from standard investigation could be analyzed.

### Whole genome sequencing (WGS)

WGS was performed for all patients as previously described [7]. WGS libraries were prepared from 1 µg of DNA with the TruSeq PCR free library prep kit following manufacturer’s recommendations (Illumina^®^, San Diego, CA, USA). 2x150bp paired-end sequences were generated on a NovaSeq 6000 instrument with 104x mean coverage (Illumina^®^, San Diego, CA, USA). Further alignment and variant filtering were performed as previously described [8]. Data were analyzed in a tumor/unmatched-normal pipeline for SNV (Strelka2), SV (Manta) and CNV (GATK). In 52/106 cases, normal tissue (*n* = 2 buccal swab, *n* = 50 CD3-positive selected T-cells) was available. Sequencing of normal control samples (mean coverage 48×) and data processing were performed using the same protocols as applied to the corresponding tumor material. Reagents for sequencing were provided by Illumina^®^, Inc. San Diego, CA.

### Whole transcriptome sequencing (WTS)

WTS was performed for all patients as previously described [9]. Stranded RNAseq libraries were constructed from ribosomal RNA-depleted RNA using TruSeq Total Stranded RNA kit (Illumina^®^, San Diego, CA, USA). 2x100bp paired-end reads were sequenced on the NovaSeq 6000 (Illumina^®^, San Diego, CA, USA) with a median of 50 million reads per sample. FASTQ generation was performed applying Illumina’s bcl2fastq software (v1.8.4). FASTQ files were preprocessed with the RNA-Seq Alignment Fast app from Illumina^®^. Data were analyzed in a tumor/unmatched-normal pipeline for fusions (Arriba/STAR-Fusion/Manta) and expression patterns. Reagents for sequencing were provided by Illumina^®^, Inc. San Diego, CA.

## Results

### Patient characteristics

In this study, 106 patients with unexplained hematological abnormalities were included. The patients had a median age of 52 years (range 3–85 years), with 44% females and 56% males (Table 1). The most common suspected diagnoses for which the patients were examined included myelodysplastic neoplasms (MDS, 18%), myeloproliferative neoplasms (MPN, 17%), plasma cell neoplasms (MGUS/MM, 9%), chronic myeloid leukemia (CML, 8%), and B-cell non-Hodgkin lymphoma (B-NHL, 8%). Another 15 patients (14%) exhibited symptoms which did not point towards a specific suspected diagnosis.

**Table 1:** Patient characteristics.

	Total cohort
	n = 106
Age (years)	
Median (range)	52 (3–85)
Sex	
Male, no. (%)	59 (56%)
Female, no. (%)	47 (44%)
Suspected diagnoses, *n* (%)	
MDS	19 (18%)
MPN	18 (17%)
MGUS/Multiple myeloma	9 (9%)
CML	8 (8%)
B-NHL	8 (8%)
T-NHL	7 (7%)
Mastocytosis	3 (3%)
MDS/MPN	2 (2%)
Other suspected hematological neoplasms	17 (16%)
Unspecific changes in blood count	15 (14%)

Details of all 106 patients are summarized regarding age, sex, and suspected diagnoses upon submission.

*CML* chronic myeloid leukemia, *NHL* non-Hodgkin lymphoma, *MDS* myelodysplastic neoplasm, *MPN* myeloproliferative neoplasm, *MGUS* monoclonal gammopathy of undetermined significance.

### WGS and WTS surpass the gold standard in 25% of cases

**Fig. 1:**
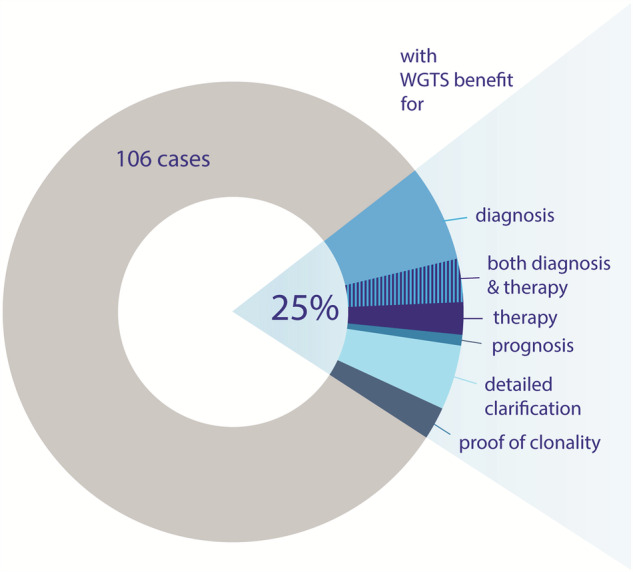
Schematic overview of the benefit of WGTS in unclear hematological cases. WGTS provided beneficial information in 25% of 106 patients with contributions to diagnosis, therapy, both diagnosis and therapy, prognosis, detailed clarification of genetic alterations or proof of clonal hematopoiesis. WGTS whole genome and transcriptome sequencing.

Compared to conventional diagnostics, WGTS predominantly identified rare SNVs not included in standard panels, CNVs too small for detection by CBA, and aberrant gene expression patterns not evaluated in standard-of-care diagnostics. Thus, WGTS analysis provided additional insights in 26 of 106 (25%) patients (Fig. 1). Of the 25% of cases where WGTS was beneficial, in 13 patients, it was possible to refine the diagnosis, and/or in 7 patients, WGTS provided valuable data influencing subsequent treatment approaches. In one patient the prognostic assessment was revised following WGTS analysis. Furthermore, in 6/26 patients WGTS contributed to detailed clarification of aberrations indicated by standard diagnostics, and in 3 patients WGTS provided the so far missing evidence of clonal hematopoiesis.

### Informative case studies to demonstrate the diagnostic value of WGS and WTS

1) A 61-year-old male patient was examined for unclear hemolytic anemia (white blood cell count (WBC): 4.1 × 10^9^/L; hemoglobin: 7.08 g/dL; platelets (PLT): 56 × 10^9^/L) with suspicion of MDS. Cytology revealed hypercellular bone marrow with an increase in megakaryopoiesis and erythropoiesis alongside maturation disorders across all three cell lineages. Notably, there were no ring sideroblasts observed and no evidence of increased blasts. Furthermore, no molecular genetic alterations were identified in a targeted myeloid panel. Applying WGS, a mutation in the gene *UBA1* (p.Ala478Ser) was detected. *UBA1* mutations are rare but have gained recognition due to their association with VEXAS syndrome. VEXAS syndrome, first identified in 2020, is characterized by therapy-refractory autoinflammation, fever, cytopenias and bone marrow dysplasia and occurs predominantly in male patients in their adult years [10, 11].

2) A 26-year-old male patient was under evaluation for suspected T-cell lymphoma or acute leukemia. Morphologically, the picture was difficult to classify based on the peripheral blood. The normocellular peripheral blood showed a left shift with 6% blasts. Erythrocytes were polychromatic and anisocytic. Normoblasts were noticeable. Thrombocytes were reduced without forming aggregates. Immunophenotyping detected mature T-lymphocytes (CD3 + CD4+: 19%; CD3 + CD8+: 16%) with regular antigen expression, NK-cells (7%) and mature B-lymphocytes (6%). For further diagnostic clarification and to exclude juvenile MDS/AML, bone marrow samples were analyzed. In those samples, a mature T-lymphoid population (44%) with expression of cyCD3 and CD2, weak expression of CD7 and lack of expression of sCD3, CD4, CD5, CD8, and TdT, as well as aberrant coexpression of CD33 was detected by immunophenotyping. The findings were consistent with a mature T-cell neoplasm and were in accordance with an aggressive T-NHL. Concurrent morphological assessment of the bone marrow trephine biopsy revealed 86.5% blastic lymphoid cells, being compatible with the diagnosis of an aggressive T-cell lymphoma. Cytogenetics showed a normal karyotype (46,XY[21]). Molecular genetic analysis identified clonal *TCRB*, *TCRD* and *TCRG* rearrangements. According to standard diagnostics, the patient presented with a T-cell neoplasm, but it remained unclear whether it was a mature T-cell lymphoma or an acute T-cell leukemia. The subsequent WGS analysis revealed an SV of chromosomes 11 and 17, resulting in a *NUP98*::*BPTF* rearrangement. Due to a lack of proliferation of the malignant cells in vitro, the aberrant clone was not detected by CBA. WTS analysis confirmed the *NUP98*::*BPTF* fusion at the RNA level. *NUP98* rearrangements occur in various hematological neoplasms, especially in children with AML and T-ALL. More than 30 fusion partners of *NUP98* have already been described. In particular, the *NUP98*::*BPTF* fusion has been detected in a patient with acute megakaryoblastic leukemia [12, 13]. In addition to the *NUP98*::*BPTF* rearrangement, a mutation in the *PTPN11* gene was detected. Although *PTPN11* is included in the standard diagnostic molecular genetic panel, WGTS enabled the detection of both aberrations in a single approach. Thus, in this patient, the inclusion of WGTS into the diagnostic strategy provided the pathological genetic aberration as marker for follow-up and supported the diagnosis of an acute leukemia with a specific genetic aberration.

3) A 34-year-old female patient with hypochromic microcytic anemia was also part of this study; the results have been published before [14]. In her case, iron deficiency, β-thalassemia, abnormal hemoglobin fractions and hemoglobin variants were excluded by standard diagnostics, whereas WGS identified deletions in chromosome 11 including a deletion of 3.8 Mb in the chromosomal region 11p15 encompassing the β-globin gene cluster, revealing a rare case of an acquired form of εγδβ-thalassemia.

### WGS and WTS can provide important insights for treatment decisions

**Fig. 2:**
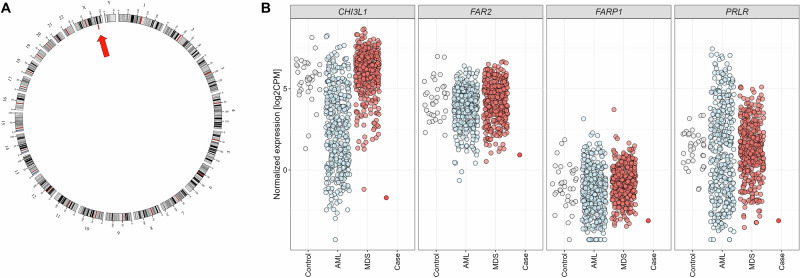
WGTS data provided evidence of clonal hematopoiesis in a patient with anemia. **A** Circos plot illustrating genomic alterations detected by WGS. The outer track runs clockwise from chromosome 1 to Y. Depicted as red bar in the inner track and highlighted by an arrow is a cytogenetically cryptic deletion spanning 4.7 Mb within the chromosomal region Xq25. This deletion encompasses the *STAG2* gene. **B** Gene expression data of *CHI3L1*, *FAR2*, *FARP1*, and *PRLR* for male control patients (gray), AML patients (light blue), and MDS patients (red-brown) with *STAG2* wild type in comparison to the patient with *STAG2* deletion (bright red). AML acute myeloid leukemia, MDS myelodysplastic neoplasm.

**Fig. 3:**
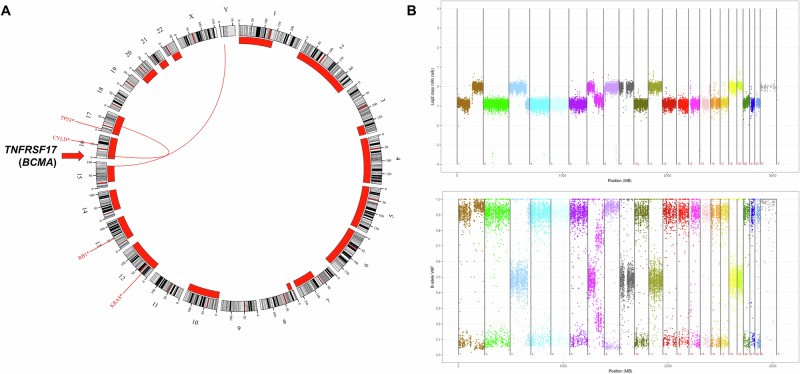
WGS analysis revealed target loss in a patient with multiple myeloma at relapse after BCMA-directed CAR T-cell therapy. **A** Circos plot with copy number variations, structural variations, and single-nucleotide variants. Outer track runs clockwise from chromosome 1 to Y. Inner track shows copy number losses >1 Mb as red bars. Red lines inside the circle represent interchromosomal structural variations. Genes with mutations (*TP53*, *CYLD*, *RB1*, *KRAS*) are depicted in red in the outer circle according to their chromosomal locations. The *TNFRSF17* gene, which encodes the antigen BCMA and is affected by a biallelic focal deletion of 512 kb in the 16p13 region, is highlighted by an arrow. **B** Illustration of copy number variation analysis according to mean log2 ratios (top) and B-allele frequencies (bottom) based on WGS data. X-axis depicts chromosomes 1 to Y from left to right. Mb megabase, WGS whole genome sequencing.

4) Given the presence of anemia (WBC: 4.66 × 10^9^/L; hemoglobin: 10.6 g/dL; PLT: 165 × 10^9^/L) in a 58-year-old male patient and clinical suspicion of MDS, gold standard diagnostics were performed but remained inconclusive. Morphological findings were suggestive of MDS but were not sufficient for diagnosing a hematological malignancy. In detail, examination revealed a normocellular peripheral blood with increased monocytes and a normocellular bone marrow exhibiting a mild increase in dysplastic erythropoiesis. Dysplastic granulopoiesis with a left shift with up to 4% blasts and occasional ring sideroblasts (<5%) were observed. Immunophenotyping also did not yield a hematological diagnosis. CBA showed a normal karyotype (46,XY[15]). Furthermore, no alterations were detected by molecular genetic analysis of a myeloid gene panel. WGS was then performed and revealed a cytogenetically cryptic deletion of 4.7 Mb in the chromosomal region Xq25 (GRCh37/hg19; chrX:123051001-127755000) (Fig. 2A), encompassing the *STAG2* gene, which encodes the STAG2 protein, a component of the cohesin complex. This finding is of particular interest, as *STAG2* mutations are well known to be recurrent in myeloid neoplasms. Mutations in the *STAG2* gene occur in 6-9% of patients with MDS, about 10% of patients with CMML and 1–6% of patients with AML, and are associated with an unfavorable prognosis in MDS and AML patients [15**–**18]. To our knowledge, *STAG2* inactivation due to a complete loss of the corresponding X-chromosomal region has not yet been described in hematological neoplasms. In a study with an AML cell line, an association of *STAG2* inactivation with an altered expression of specific genes was shown [19]. Based on WTS, we were also able to observe the described deregulated expression of genes such as *CHI3L1*, *FAR2*, *FARP1*, and *PRLR* (Fig. 2B). Interestingly, CHI3L1 plays an important role in the MAPK signaling pathway [20]. Smith et al. have shown that the reduced expression of several key players of the MAPK signaling pathway has a functional effect and results in *STAG2*-depleted cases being more sensitive to MEK inhibitors, making this a potential treatment option [19]. Considering the comprehensive findings of the WGTS analysis and the resulting evidence of clonal hematopoiesis along persistent anemia, a clonal cytopenia of undetermined significance (CCUS) was diagnosed.

5) A 64-year-old female patient presented with an acute leukemia though specific classification remained unclear (WBC: 209 × 10^9^/L; hemoglobin: 9.6 g/dL; PLT: 244 × 10^9^/L). Cytology indicated predominantly lymphatic blasts, which were consistent with an observed involvement of lymph nodes and the stomach. Immunophenotyping showed expression of CD7, CD34, TdT, co-expression of CD33 and CD117 and weak expression of CD13 and cCD3 and could therefore correspond to the phenotype of an early T-cell precursor ALL (ETP-ALL). Due to the weak expression of cCD3, a possible differential diagnosis was AML with minimal differentiation. Molecular genetics identified mutations in the genes *ASXL1*, *DNMT3A*, *NRAS* and *TET2*. By CBA and FISH analysis, a translocation of chromosomes 4 and 12 was detected (46,XX,t(4;12)(q12;p13)[20]), that involved the chromosomal region of the *PDGFRA* gene. As *PDGFRA* rearrangements are not common in ETP-ALL or AML, WGTS was indicated for detailed genetic clarification. In accordance with the 4;12-translocation, an SV of chromosomes 4 and 12 was detected, which affected the *ETV6* gene on chromosome 12. On chromosome 4, the chromosomal breakage was identified to be located between the genes *CHIC2* and *PDGFRA*. Therefore, a rearrangement in the region of the *PDGFRA* gene could be confirmed, but there was no indication of a *PDGFRA* fusion. Subsequently, the implications of the rearrangement were further analyzed based on WTS data, revealing a significantly elevated *PDGFRA* expression. Of importance, the identification of a *PDGFRA* rearrangement and the associated strong *PDGFRA* expression pointed to sensitivity to therapy with tyrosine kinase inhibitors.

6) In a 41-year-old male patient with multiple myeloma (MM) at early relapse after BCMA-directed CAR T-cell therapy, FISH analysis on purified CD138-positive cells from a bone marrow aspirate showed high-risk disease with the detection of a 17p deletion [21]. Because of uncertainty about the optimal subsequent therapeutic strategy, WGTS analysis was performed and revealed a *TP53* mutation as an additional high-risk feature and hyperhaploidy with numerous chromosomal losses, which is also associated with an unfavorable prognosis in MM [22]. Moreover, mutations in the genes *KRAS*, *RB1* and *CYLD* were detected. These complex genetic aberrations were consistent with an advanced disease. Most relevant, WGS also revealed a monosomy 16 and a focal deletion of 512 kb in the 16p13 region of the remaining chromosome 16 (GRCh37/hg19; chr16:12056001-12568000) including the *TNFRSF17* gene (Fig. 3). *TNFRSF17* encodes the antigen BCMA, which is used as target in several immunotherapies for MM patients and biallelic genomic loss of *TNFRSF17* has been described as resistance mechanism to BCMA-targeted immunotherapies [23, 24]. In accordance with the complete loss of *TNFRSF17*, WTS analysis showed significantly reduced *TNFRSF17* expression. In this case, WGTS provided clinically highly relevant information for further treatment decisions, as other BCMA-targeted therapies cannot be considered as an option due to target loss.

### Beneficial impact of WGS and WTS on prognostic assessment

**Fig. 4:**
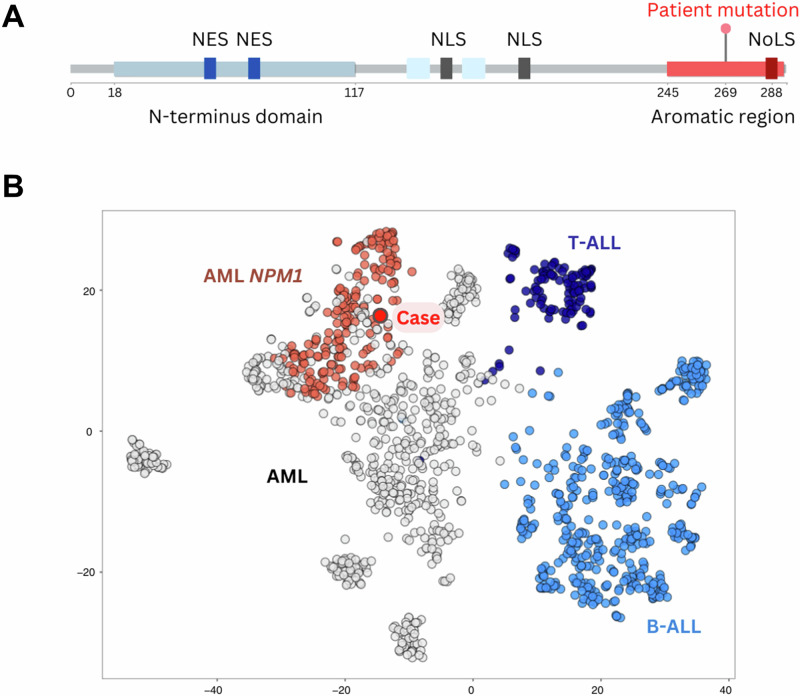
Identification of a rare *NPM1* mutation by WGTS in an AML patient. **A** Illustration of the mutation in the *NPM1* gene. The panel shows the mutation located in exon 10 of the *NPM1* gene (p.Ile269Alafs*7), which is located in the highly conserved aromatic region, akin to Trp288 and Trp290 that are found in the NoLS. NES nuclear export signal, NLS nuclear localization signal, NoLS nucleolar localization signal. **B** Clustering of the case by gene expression analysis. The patient is highlighted in bright red and clusters together with *NPM1* mutated AML patients (red-brown). AML acute myeloid leukemia, WGTS whole genome and transcriptome sequencing.

7) A 51-year-old male patient was examined because of recurrent infections. Clinical findings included a WBC of 5.3 × 10^9^/L, hemoglobin at 11.5 g/dL and platelets at 309 × 10^9^/L. According to WHO, cytomorphology suggested the diagnosis of an AML with maturation (blasts 24.5%; segmented granulocyte 35.5%; monocytes 1.5%) and the immunophenotype corroborated the diagnosis of an AML (CD133(+)/CD34-/CD117+/CD13-/CD45(+)). In CBA a normal karyotype (46,XY[20]) was observed and molecular genetics did not reveal any aberrations, resulting in an assignment of the patient to the intermediate risk group [25]. However, WGTS analysis identified a previously undetected mutation in the *NPM1* gene in exon 10 (p.Ile269Alafs*7; ENST00000296930), which differs from the common hotspot region in exon 11 that is routinely analyzed in AML patients. In addition, gene expression analysis showed clustering of this case with the subgroup of AML patients with *NPM1* mutation (Fig. 4). These results imply a possible analogous function of the identified variant with known hotspot *NPM1* mutations leading to dysfunction of NPM1 due to mislocalization in the cytoplasm. With the inclusion of WGTS analysis, the identification of an *NPM1* mutation without *FLT3*-ITD mutation indicated reclassification from the intermediate to the favorable risk category according to the 2022 European LeukemiaNet recommendations [25].

### Time to process WGTS analysis

In this study with individual patient cases, WGTS analysis was completed and reported within a median of 27 days (range 9–66 days). Deviations from the average were primarily due to outliers, where additional material had to be requested or repeat runs were necessary. According to current guidelines, gold standard diagnostic methods were prioritized, and WGTS analysis is not yet established in routine diagnostic workflows. Although expedited diagnostics via WGTS are already feasible within 5–7 days, for this study the consolidation of samples into batches for economic efficiency occasionally extended the turnaround time. Therefore, the duration stated above does not reflect the potential for a fast and holistic analysis inherent to WGTS approaches.

## Discussion

Diagnosing hematological neoplasms is complex and requires a comprehensive approach. Despite applying all gold standard diagnostic methods in alignment with the latest guidelines, certain cases remain unsolved. This unmet need for additional options in the diagnostic toolbox raises the question whether the application of WGS and WTS can provide crucial information in such challenging cases.

Here, we present a head-to-head comparison of current gold standard diagnostics with WGTS in unclear but suspicious hematological cases. In this study, 25% of cases benefited from the additional diagnostic information provided by WGTS, underlining its potential to positively impact clinical decision-making. Until now, WGS and WTS have predominantly served as research tools. However, an increasing number of studies are retrospectively investigating the diagnostic utility of genome sequencing in specific hematological neoplasms [26–29]. The prospective design of our study is crucial, focusing exclusively on cases with unsolved diagnoses or unclear conditions even after application of all established diagnostic analyses, where urgent clinical decisions were needed in real-time. By implementing WGTS in the real-world setting, we demonstrated its potential to swiftly provide critical insights that influence patient diagnosis, prognosis, and treatment options. WGTS represents a significant step forward by streamlining the diagnostic process. Extracting DNA and RNA from a single sample eliminates the need for multiple, consecutive tests as it is common in conventional diagnostics. Therefore, material availability is in most cases not a limiting factor compared to standard diagnostics, because WGTS offers a one-step solution for genomic and transcriptomic analysis. At the same time, WGTS enables the rapid implementation of new research findings into clinical diagnostics, as exemplified by the detection of *UBA1* mutations associated with VEXAS syndrome [10].

Furthermore, its comprehensive approach facilitates the identification of novel gene fusions, expanding the spectrum of detectable genetic alterations beyond what is possible with advanced but guideline-conforming conventional methods.

Looking ahead, the relevance of genetic classification in hematological neoplasms is expected to increase significantly, given that genetic information is becoming more and more essential for prognostication, MRD assessment, and the implementation of precision medicine-based strategies. Accurate genetic profiling enables clinicians to stratify patients according to risk, select the most appropriate and personalized therapies, and monitor diseases more precisely using patient-specific MRD markers. This improves outcomes and can help ensure cost-effective care. WGS enables the identification of all genomic aberrations, while WTS provides crucial insights into gene expression and the functional impact of these aberrations. This is particularly important, as not all genetic alterations are necessarily expressed or functionally relevant. WTS can reveal active transcription of a mutation or fusion gene, thereby highlighting functionally significant aberrations that may serve as therapeutic targets [30]. Furthermore, the clonal hierarchy is becoming a key factor in therapy selection and disease monitoring. WGS enables reconstruction of the clonal architecture, identifying both early (founder) and late (subclonal) events, while WTS can confirm which of these events are transcriptionally active [30, 31]. The integration of both technologies allows for a more nuanced understanding of disease biology and ensures that targeted therapies are directed against the most clinically relevant, functionally active clones. Our case of MM with BCMA-loss illustrates that the timely use of WGTS can identify both primary cancer diagnoses and secondary resistance mechanisms following targeted therapies [32]. With the widespread adoption of such strategies in MM and other cancers, detection of resistance-related genomic events is crucial to optimize patient outcomes and avoid ineffective treatments and their associated costs.

Our analyses also found - even with all diagnostic efforts - 80 cases with no gain of additional diagnostic information provided by WGTS. This outcome should not be interpreted as a shortcoming of WGTS technologies per se, but rather as an indication of the extensive yet-to-be-explored complexities of the human genome. A considerable number of variants detected by WGTS were classified as being variants of uncertain significance (VUS), mainly because they could not be definitively associated with known genetic markers of hematological neoplasms. Functional large-scale studies are needed to close this knowledge gap in our understanding of the human genome [33]. The evaluation of cases in this study is further constrained by focusing on somatic mutations, underscoring the importance of the incorporation of germline mutations and hereditary aspects into a comprehensive diagnostic strategy.

While WGTS represents a significant advancement in our ability to uncover genetic and transcriptomic alterations, it is not a universal solution for all diagnostic challenges. Its utility is influenced by factors such as the sensitivity of the sequencing technology and the specific clinical context of each patient. For patient samples harboring low-abundance or subclonal mutations, such as those potentially indicative of early-stage lymphomas or other hematological neoplasms, the sensitivity of WGTS analysis may not always be sufficient to detect these abnormalities. This limitation can lead to the exclusion of cases where small, yet clinically significant, clones fall below the detection threshold of the sequencing technology. The use of WGTS is also limited by the need for extensive data processing and storage capabilities. The vast amount of WGTS data requires substantial computational resources for analysis and secure, long-term storage solutions, which also demand advanced IT infrastructure and expertise [34].

In addition to clinical and technical limitations, economic and ethical considerations are crucial when integrating WGTS into routine diagnostics. The financial implications of repeated standard diagnostics versus targeted genomic testing are complex: The cost of WGS varies significantly, with estimates from an Australian micro-costing study between US$2006–3347 (€1700–2837 in 2018) per case [35], while an analysis from the UK estimated £6841–7050 (€7802–8040 in 2019) per patient [36]. A Swedish study analyzing the costs for acute leukemia calculated average WGS costs of €3472 and €2671 in 2024, based on an annual throughput of 2500 or 7500 analyses, respectively [37]. However, it is challenging to compare these studies due to rapidly changing sequencing costs, the absence of standardized WGS workflows, and variations in micro-costing methodologies [37]. In contrast, panel-based testing varies between US$260–3600 (€240–3300 in 2023) [38] and costs for exome sequencing between £382–3592 (€431–4059 in 2018) [39]. The major cost drivers for WGS include consumables, accounting for approximately 76% of total costs, followed by staff (9%). Equipment, bioinformatics, and data maintenance accounted for less than 10% of total costs [37]. A health-economic model with non-small-cell lung cancer patients suggested WGS could be cost-effective if the price per patient dropped below €2000 and if it identified more actionable findings (2.7%) than current diagnostics [40]. As sequencing reagent and instrument costs continue to decline, WGS may soon approach the range of traditional genetic assays [26, 37, 40, 41]. Given these financial considerations, our research suggests not only a lowering of the burden of uncertainty among patients and physicians, no wrong diagnosis or treatment, but also the potential for cost savings through the strategic, stepwise implementation of WGTS. However, the higher initial costs of genomic testing raise concerns about accessibility and equity, necessitating a balanced approach that weighs both economic and ethical factors. We here provide data that the additional diagnostic value provided by WGTS in 25% of cases justifies its broader application, aligning with medical ethics that prioritize patient welfare and informed consent.

## Conclusion

In summary, WGTS analysis has significant clinical relevance in selected cases in hematology and represents a beneficial addition to conventional diagnostics from a medical as well as health economic and ethical point of view. It is imperative that healthcare systems and policy-makers consider these insights to optimize diagnostic strategies, ensuring that they not only enhance patient outcomes but also do so in a manner that is financially sustainable and ethically responsible. Moving forward, it will be crucial to fill the gaps in the knowledge of disease-related genetics to unlock the full potential of genomic diagnostics. This endeavor will not only require technological advancements in the laboratory and in bioinformatic tools but also collaborative efforts to expand our understanding of the human genome, ensuring that genomic diagnostics will make a significant contribution to personalized medicine and improved patient care.

## Supplementary information

Supplementary Table

## Data Availability

The datasets generated during and/or analyzed during the current study are available from the corresponding author on reasonable request.
